# *TERT* promoter mutations in penile squamous cell carcinoma: high frequency in non-HPV-related type and association with favorable clinicopathologic features

**DOI:** 10.1007/s00432-021-03514-9

**Published:** 2021-02-26

**Authors:** Sang Kyum Kim, Jang-Hee Kim, Jae Ho Han, Nam Hoon Cho, Se Joong Kim, Sun Il Kim, Seol Ho Choo, Ji Su Kim, Bumhee Park, Ji Eun Kwon

**Affiliations:** 1 Department of Pathology, Severance Hospital, Yonsei University College of Medicine, Seoul, Republic of Korea; 2 Department of Pathology, Ajou University School of Medicine, 164, Worldcup-ro, Yeongtong-gu, Suwon, 16499 Republic of Korea; 3 Department of Urology, Ajou University School of Medicine, Suwon, Republic of Korea; 4 Office of Biostatistics, Medical Research Collaborating Center, Ajou Research Institute for Innovation, Ajou University Medical Center, Suwon, Republic of Korea; 5 Department of Biomedical Informatics, Ajou University School of Medicine, Suwon, Republic of Korea

**Keywords:** *TERT* promoter, Penile cancer, Human papillomavirus, Telomerase

## Abstract

**Purpose:**

Penile carcinoma is a rare malignant neoplasm with a largely unknown molecular pathogenesis. Telomerase reverse transcriptase promoter (*TERT-*p) mutations have been detected in several types of human malignancies. The aim of this study was to investigate the presence of *TERT-*p mutations in penile squamous cell carcinomas (SCCs) and their associations with clinicopathologic features.

**Methods:**

In this retrospective study, Sanger sequencing was performed to detect *TERT-*p mutations in formalin-fixed paraffin-embedded tissue samples from 37 patients with penile SCC, 16 patients with cutaneous SCC, and 4 patients with non-neoplastic penile/skin tissue. The expression of p16^INK4a^ and Ki-67 was investigated via immunohistochemistry. Associations of *TERT-p* mutation with clinicopathological factors, immunohistochemical results, and clinical outcome were statistically analyzed.

**Results:**

Recurrent *TERT-p* mutations were identified in 18 out of 37 (48.6%) penile SCCs, including all 3 carcinoma in situ cases. *TERT-p* mutations were significantly more frequent in non-human papilloma virus (HPV)-related penile SCC types than in non-HPV-related penile SCC based on both histologic classification and p16^INK4a^ immunoreactivity. Furthermore, *TERT-p* mutation was associated with a low histologic grade, low mitotic count, absence of necrosis, low Ki-67/MIB-1 labeling index, and absence of lymph node or distant metastasis.

**Conclusion:**

Our study shows *TERT*-p mutations are the most frequent somatic mutations in penile SCC. In addition, *TERT*-p mutations are far more frequent in non-HPV-related penile SCC than in HPV-related penile SCC, indicating *TERT*-p mutations may have a role in tumorigenesis distinct from HPV-related penile SCC.

**Supplementary Information:**

The online version contains supplementary material available at 10.1007/s00432-021-03514-9.

## Introduction

Penile carcinoma is a rare malignant neoplasm with an incidence of 1–4 per 100,000 in developing countries and is even rarer in developed countries (Rubin et al. 2001). Squamous cell carcinoma (SCC) and its histologic variants account for approximately 95% of all cases of penile carcinoma (Suarez-Bonnet et al. 2018). Pathologically, penile carcinomas are classified into two distinct groups based on clinicopathologic characteristics and an association with HPV infection: human papilloma virus (HPV)-related and non-HPV-related type (Mannweiler et al. 2013). HPV high-risk (HPV-HR) DNA is detected in 20–80% of penile carcinomas, and plays a role in the carcinogenesis of HPV-related penile SCC (Spiess et al. 2016). The detection rates of HPV DNA vary depending on histologic subtypes (Cubilla et al. 2016). Unlike SCC of the uterine cervix, which is caused by HPV in 100% of cases, only about 1/3–1/2 of penile carcinomas are caused by transforming HPV infection. The non-HPV-related penile SCC develops in the background of chronic inflammatory skin diseases such as lichen sclerosus and lichen planus. HPV-HR16 is known to be the most prevalent HPV DNA type in HPV-related penile SCC (Rubin et al. 2001). The viral oncogenes of HPV, E6 and E7, bind to the tumor-suppressor proteins p53 and RB, respectively, leading to their inactivation, thereby affecting the cell cycle and apoptosis and resulting in unchecked replication of DNA and continued cell proliferation (Spiess et al. 2016). Consequently, p16^INK4a^ protein, a cyclin-dependent kinase inhibitor, accumulates, which can be identified by p16^INK4a^ immunohistochemistry (Aumayr et al. 2013).

Along with technological advances in the past decade, genetic alterations in cancers have been extensively disclosed, ushering in a new era of precision medicine in cancer treatment (Baudino 2015). However, this has not been the case for penile cancer, as the underlying molecular pathogenesis is yet to be elucidated, which is most likely due to the rarity of penile SCC. Accordingly, no standardized treatment or personalized medicine has been established for penile cancer (Kim et al. 2018).

The telomerase reverse transcriptase (*TERT*) gene encodes the catalytic subunit of telomerase that is responsible for telomere lengthening at chromosomal ends (Vinagre et al. 2014). Normal somatic cells and benign tumor cells lack telomerase activity, whereas telomerase is active in germ cells and stem cells of self-renewing tissues (Gunes and Rudolph 2013), and is found to be reactivated in up to 90% of cancer cells (Kyo et al. 2008). Reactivation of telomerase maintains the telomere length and enables cancer cells to evade senescence resulting from telomere shortening. Novel recurrent somatic mutations in the core promoter region of *TERT* were recently identified in ~ 70% of melanoma samples examined by next-generation sequencing, which have now become the most frequently detected mutation in malignant melanoma (Horn et al. 2013; Huang et al. 2013). Subsequently, these mutations were also detected at high frequencies in several other human malignancies, including thyroid carcinoma, glioma, bladder carcinoma, hepatocellular carcinoma, and various types of non-melanoma skin cancers such as SCC and basal cell carcinoma (Arita et al. 2013; Allory et al. 2014; Chen et al. 2014a, b; Griewank et al. 2013; Liu et al. 2013a, b; Populo et al. 2014; Scott et al. 2014). By contrast, *TERT* promoter (*TERT-*p) mutations have not been detected or have been detected at very low frequency in malignant tumors of other organs (Cheng et al. 2015; Huang et al. 2013; van Nistelrooij et al. 2014). In addition, the clinical relevance of these mutations has been highlighted based on significant associations with poor patient outcome or adverse clinicopathologic parameters in various cancers (Chen et al. 2014a, b; Kinde et al. 2013; Griewank et al. 2014; Melo et al. 2014; Qu et al. 2014; Simon et al. 2015). *TERT*-p mutations create de novo binding sites for E-twenty-six (ETS) transcription factors (CCGGAA) within the *TERT*-p region. An in vitro luciferase assay also showed that these mutations resulted in a two- to fourfold increase of TERT promoter activity (Huang et al. 2013). Despite continuing research on *TERT*-p mutation in human cancers, including a recent study describing the varying frequency of *TERT*-p mutation in SCCs arising from various organs (Cheng et al. 2015), *TERT*-p mutation in penile SCC has not been investigated to date.

Therefore, the aim of the present study was to investigate the presence of *TERT-*p mutations in penile SCC, and their associations with clinicopathologic parameters, along with p16^INK4a^ and Ki-67/MIB-1 expression.

## Methods

### Case selection

The study was approved by the Institutional Review Boards (IRB) of Ajou University School of Medicine and Yonsei University Health System, and performed in accordance with the Declaration of Helsinki. Informed consent was waived by the IRB for this retrospective study. Thirty-seven formalin-fixed, paraffin-embedded penile carcinoma tissues surgically resected from patients in the time period between 2000 and 2017 were retrieved from the archives of the Department of Pathology of Ajou University School of Medicine (23 cases) and the Department of Pathology of Yonsei University College of Medicine (14 cases) based on the availability of tissue blocks or slides for histopathologic analyses and DNA extraction. Among the 37 cases, there were three cases of carcinoma in situ (CIS) included. In addition, 16 cases of cutaneous SCC and 4 samples of non-tumor skin or penile tissue were used as positive and negative controls, respectively, for *TERT*-p sequencing.

### DNA extraction and direct sequencing

The tumors were manually dissected from 10-μm sections of formalin-fixed, paraffin-embedded tissues. Genomic DNA was extracted using the QIAamp kit (Qiagen, Hilden, Germany) according to the manufacturer’s instructions. Polymerase chain reaction (PCR) amplification of the *TERT* promoter region was performed using the primer pair h*TERT*_F (CAC CCG TCC TGC CCC TTC ACC TT)/h*TERT*_R (CAG CGC TGC CTG AAA CTC), generating an expected 304-bp product. The cycling conditions for PCR amplification were 95 ℃ for 2 min for denaturation, and 35 cycles of 95 ℃ denaturation for 30 s, 60 ℃ annealing for 30 s, and 72 ℃ elongation for 40 s. PCR products were confirmed by gel electrophoresis. Direct sequencing of both strands was performed using a BigDye terminator v3.1 cycle sequencing kit (Applied Biosystems, Foster City, CA, USA) on an ABI 3500XL genetic analysis system (Applied Biosystems).

### Clinicopathologic features

All hematoxylin and eosin-stained slides from the tumors were reviewed independently by two pathologists who were blinded to other clinical and genetic information. Clinical data were collected from the medical records. The factors investigated were as follows: histologic subtype, grade, presence and type of penile intraepithelial neoplasia (PeIN), lymphovascular invasion, perineural invasion, ulceration, tumor thickness, necrosis, mitotic count, koilocytosis, acantholysis, tumor-infiltrating lymphocytes, intraepithelial neutrophilic microabscess, peripheral tumor budding (Shimizu et al. 2018), tumor size, gross type, patient age, stage, lymph node or distant metastasis, and patient survival (Online Resource 1–3). Histologic subtype was classified into HPV-related type and non-HPV-related type according to the World Health Organization guidelines 2016 (Cubilla et al. 2016). A mixed HPV-related and non-HPV-related type was considered a HPV-related type for statistical analyses. The mitotic count was calculated from 10 contiguous high-power fields located in the most mitotically active tumor region.

### Immunohistochemistry for p16^INK4a^ and Ki-67

Immunohistochemical staining for p16^INK4a^ and Ki-67 was performed on one representative block of all samples on a BenchMark XT autostainer (Ventana Medical Systems, Tucson, AZ, USA) according to the manufacturer’s protocol using a ready-to-use mouse monoclonal p16^INK4a^ antibody (CINtec p16 Histology, clone E6H4, Sedona, USA) and an anti-human mouse monoclonal Ki-67 antibody (1:100, clone MIB-1, DAKO, Copenhagen, Denmark). Only strong continuous staining of p16^INK4a^ was considered positive. Weak or spotty, patchy, and discontinuous staining was considered negative (Katzenellenbogen 2017a, b). For the Ki-67 stain, any distinct nuclear staining was recorded as positive. The Ki-67 (MIB-1) labeling index, defined as the percentage of positively stained tumor cells, was measured by computer-assisted manual counting of at least 1000 tumor nuclei from the area of maximal labeling using Image-Pro Plus 4.5 software.

### Statistical analyses

Statistical analyses were performed using SPSS v22.0 (SPSS Inc., Chicago, IL, USA). The relation between *TERT*-p mutation and clinicopathologic parameters was evaluated using the *χ*^2^ test or Fisher’s exact test and the Mann–Whitney *U* test for categorical and continuous variables, respectively. Kaplan–Meier survival analysis and the log-rank test were used to analyze the prognostic effect of *TERT*-p mutation in terms of both disease-free survival (DFS) and overall survival (OS). Univariate and multivariate regression analyses were performed using the Cox proportional hazards model. A *P* value < 0.05 was considered significant. We conducted a power analysis for determining an appropriate sample size in Cox proportional hazard regression analysis using PASS v14.0.14.

## Results

### Study cohort

The age at diagnosis for the included patients was 65.97 ± 12.18 years (range 40–87 years). Of the 37 penile SCCs, 17 were HPV-related type and 20 were non-HPV-related type. Follow-up data were available for all 37 cases, and the follow-up period ranged from 4 months to 13.8 years (median 80.47 months). Seven patients died of the disease (Table 1).

**Table 1:** Summary of patient characteristics

Characteristics	*N* = 37
Age at diagnosis (years)	
Mean ± SD	65.97 ± 12.18
Range	40–87
Tumor size (cm)	
Mean ± SD	3.59 ± 2.43
Range	1–14
Histologic type (no. of patient)	
HPV-related	17
Non-HPV-related	20
AJCC stage (no. of patient)	
I	8
II	18
III	5
IV	6
Grade (no. of patient)	
1 (well differentiated)	10
2 (moderately differentiated)	9
3 (poorly differentiated)	18
Gross type (no. of patient)	
Superficial spreading^a^	8
Verrucous	13
Vertical	16
Surgery (no. of patient)	
Total penectomy	6
Partial penectomy	28
Excision	3
Lymph node biopsy or dissection	14
Adjuvant therapy	
Chemotherapy, only	3
Radiotherapy, only	2
Chemoradiotherapy	4
Follow-up duration (month)	
Median	80.47
Range	4–166
Metastasis (no. of patient)	
Lymph node	10
Distant metastasis	7
Bone	3
Lung	4
Clinical outcome (no. of patient)	
Alive	22
Died of disease	7
Died of other cause	5

*SD* standard deviation, *AJCC* the American Joint Committee on Cancer

^a^The three cases of carcinoma in situ were included

### *TERT-*p mutations are frequent in penile SCC

Of the 37 penile SCCs, including the 3 cases of CIS, 18 cases (48.6%) harbored *TERT*-p mutations, including c.-146 C > T, c.-124C > T, and c.-124 C > A. All three CIS tumors harbored the c.-146 C > T mutation. Ten (62.5%) of the 16 skin SCCs had *TERT*-p mutations, which were located at positions c.-146 C > T, c.-124C > T, and c.-139_-138CC > TT (Online Resource 4). The four negative control samples (non-neoplastic penile or skin tissue) all showed the wild-type *TERT*-p sequence (Table 2).

**Table 2:** *TERT* promoter mutations identified in penile squamous cell carcinoma and control tissues

Tissue	*N* (%)	*TERT*-p mutant					*TERT*-p wild
		Overall	c.-146 C > T	c.-124 C > T	c.-124 C > A	c.-139_-138CC > TT	
Penile SCC invasive	34 (100%)	15 (44.1%)	11 (32.3%)	3 (8.8%)	1 (2.9%)	0	19 (55.8%)
Penile SCC in situ	3 (100%)	3 (100%)	3 (100%)	0	0	0	0
Penile SCC in situ and invasive	37 (100%)	18 (48.6%)	14 (37.8%)	3 (8.1%)	1 (2.7%)	0	19 (51.3%)
Skin SCC	16 (100%)	10 (62.5%)	8 (50.0%)	1 (6.2%)	0	1 (6.2%)	6 (37.5%)
Pen NT^a^	2 (100%)	0	0	0	0	0	2 (100%)
Skin NT^a^	2 (100%)	0	0	0	0	0	2 (100%)

*No* number, *Pen* penile, *NT* non-tumor tissue, *SCC* squamous cell carcinoma

^a^Negative control

### Correlation of *TERT*-p mutation status with clinicopathologic parameters

To explore potential associations between *TERT-*p mutation status and clinicopathologic parameters, statistical analyses were performed on the 24 invasive SCCs, excluding the three CIS cases. *TERT*-p mutations were more frequent in non-HPV-related type than in HPV-related type penile SCCs (13/20, 86.7% vs 2/14, 13.3%; *p* = 0.005). In line with this finding, *TERT-*p mutations correlated with the presence of differentiated PeIN, which is a precursor lesion associated with non-HPV-related type penile SCC (*p* = 0.005). *TERT*-p mutations were also more frequent in tumors with a lower histologic grade (*p* = 0.036), lower mitotic activity (*p* = 0.001), absence of necrosis (*p* = 0.045), larger tumor size (*p* = 0.045), and absence of lymph node or distant metastasis (*p* = 0.020) (Table 3).

**Table 3:** Associations between *TERT* promoter mutation and clinicopathologic parameters in 34 patients with invasive penile squamous cell carcinoma

Parameters	*TERT*-p wild (*N* = 19)	*TERT*-p mutant (*N* = 15)	*p* value
Histologic subtype			0.005*
HPV-related	12 (63.2%)	2 (13.3%)	
Non-HPV-related	7 (36.8%)	13 (86.7%)	
Histologic grade			0.036*
WD	3 (15.8%)	7 (46.7%)	
MD	5 (26.3%)	4 (26.7%)	
PD	11 (57.9%)	4 (26.7%)	
Acantholysis			0.462
Absent	15 (78.9%)	10 (66.7%)	
Present	4 (21.1%)	5 (33.3%)	
Lymphovascular invasion			0.409
Absent	10 (52.6%)	10 (66.7%)	
Present	9 (47.4%)	5 (33.3%)	
Perineural invasion			0.697
Absent	13 (68.4%)	12 (80.0%)	
Present	6 (31.6%)	3 (20.0%)	
Koilocytosis			0.260
Absent	10 (52.6%)	5 (33.3%)	
Present	9 (47.4%)	10 (66.7%)	
Mitosis (/HPF)			
Mean ± SD	9.02 ± 6.92	2.68 ± 1.71	
≤ 8	10 (52.6%)	15 (100%)	0.001*
> 8	9 (47.4%)	0 (0%)	0.002*
Tumor thickness (cm)			0.300
< 1.2	11 (57.9%)	6 (40.0%)	
≥ 1.2	8 (42.1%)	9 (60.0%)	
Necrosis			0.045*
Absent	7 (36.8%)	11 (73.3%)	
Present	12 (63.2%)	4 (26.7%)	
Tumor infiltrating lymphocytes			0.476
Absent to non-brisk	11 (57.9%)	11 (73.3%)	
Brisk	8 (42.1%)	4 (26.7%)	
Ulceration			0.300
Absent	8 (42.1%)	9 (60.0%)	
Present	11 (57.9%)	6 (40.0%)	
Peripheral budding			0.968
Absent to focal (≤ 10%)	10 (52.6%)	8 (47.4%)	
Diffuse (> 10%)	9 (53.3%)	7 (46.7%)	
Intraepithelial microabscess			0.152
Absent	9 (47.4%)	3 (20.0%)	
Present	10 (52.6%)	12 (80.0%)	
T stage			0.679
1	7 (36.8%)	3 (20.0%)	
2	7 (36.8%)	9 (60.0%)	
3	5 (26.3%)	3 (20.0%)	
AJCC stage			0.128
I, II	11 (57.9%)	13 (86.7%)	
III, IV	8 (42.1%)	2 (13.3%)	
LN or distant metastases			0.020*
Absent	10 (52.6%)	14 (93.3%)	
Present	9 (47.4%)	1 (6.7%)	
Tumor size (cm)			0.045*
≤ 3	12 (63.2%)	4 (26.7%)	
> 3	7 (36.8%)	11 (73.3%)	
Age, years			
Mean ± SD	67.47 ± 13.24	64.27 ± 13.04	0.560
PeIN			0.005*
Non-HPV-related type	7 (36.8%)	13 (86.7%)	
HPV-related type	12 (63.2%)	2 (13.3%)	

*HPV* human papilloma virus, *HPF* high-power field, *WD* well differentiated, *MD* moderately differentiated, *PD* poorly differentiated, *AJCC* the American Joint Committee on Cancer, *LN* lymph node, *PeIN* penile intraepithelial neoplasia

*p < 0.05

### Correlation of *TERT* mutation status with p16^INK4a^ and MIB-1 immunohistochemistry

**Fig. 1:**
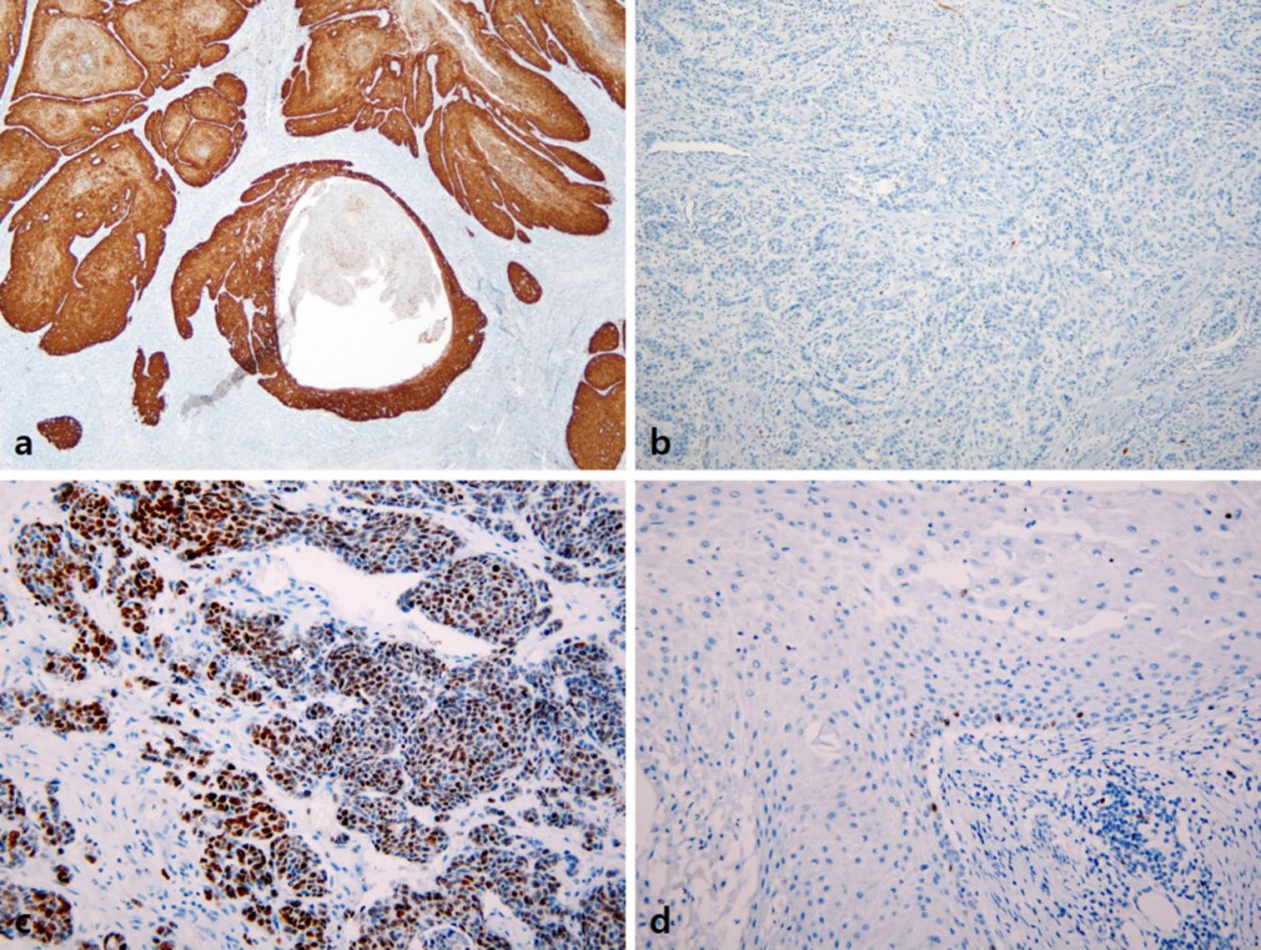
Immunohistochemical staining for p16^INK4a^ and MIB-1 in penile SCC. **a** Diffuse strong positivity for p16^INK4a^ in *TERT*-p wild-type penile SCC. **b** p16^INK4a^ negativity in *TERT*-p-mutated penile SCC. **c** High MIB-1 labeling index. **d** Low MIB-1 labeling index

p16^INK4a^ positivity was more frequent in *TERT*-p wild-type tumors (12/19, 63.2%) than in *TERT*-p mutant tumors (1/15, 6.7%; *p* = 0.001), which is consistent with the above result that *TERT*-p mutations were more frequent in non-HPV-related type than in HPV-related type penile SCC. In addition, the MIB-1 labeling index was significantly higher in *TERT*-p wild-type tumors (45.26 ± 19.38) than in *TERT*-p mutant tumors (31.80 ± 11.89; *p* = 0.014; Fig. 1).

### Effects of *TERT*-p mutations on prognosis of patients with penile SCC

**Fig. 2:**
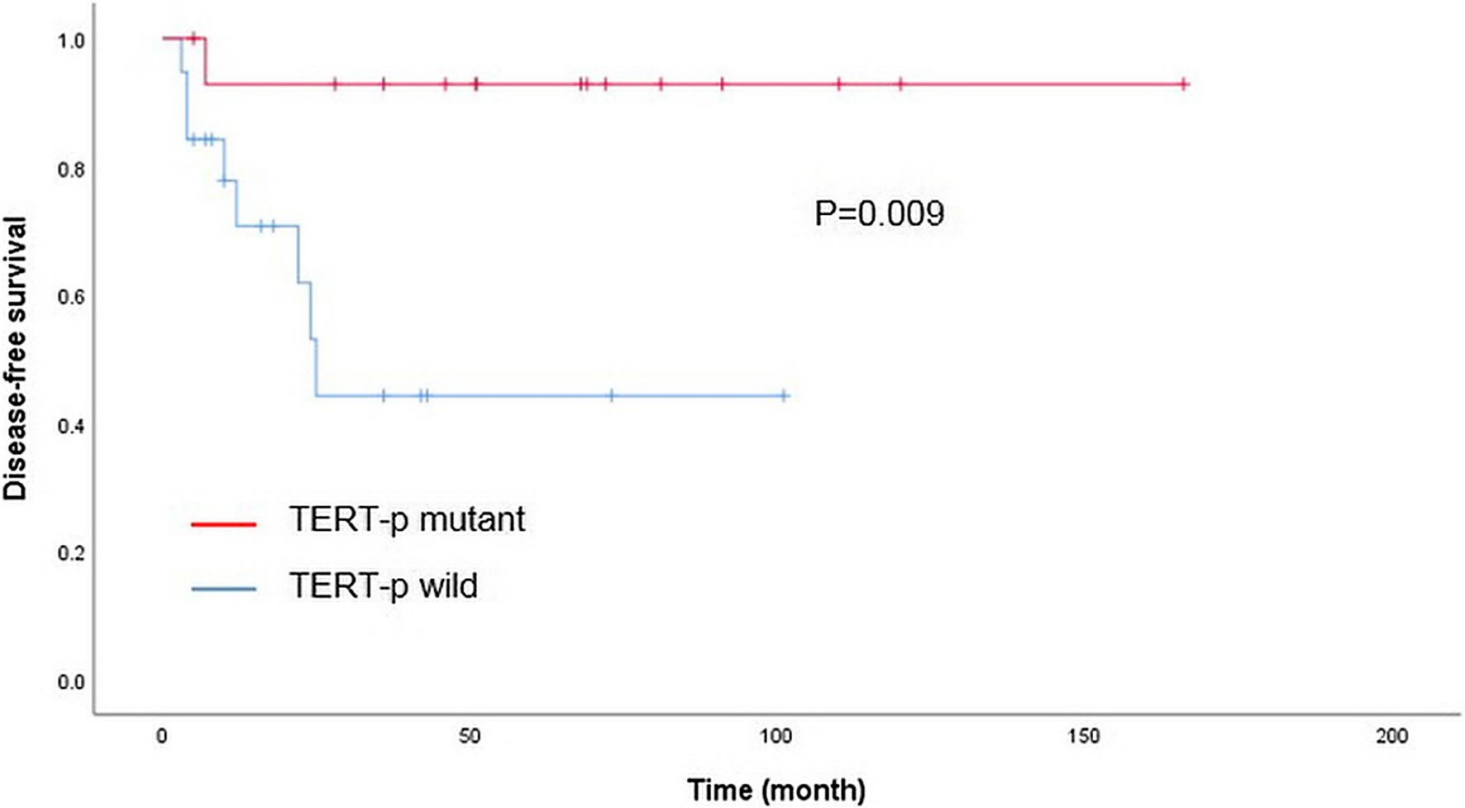
Kaplan–Meier analysis of the impact of *TERT*-p mutation on disease-free survival of patients with penile SCC

We performed a power analysis to estimate the adequacy of sample size for Cox regression analysis in our study. A sample size of 34 subjects (wild group = 19, mutant group = 15) was determined to be optimal with 94.90% statistical power, a significance level of *p* < 0.05, and hazard ratio = 0.101(= 1/9.86). As shown in Fig. 2, patients with *TERT*-p-mutated tumors showed significantly longer DFS compared with that of patients with *TERT*-p wild-type tumors (*p* = 0.009). However, there was no difference in OS between patients with *TERT*-p-mutated and wild-type tumors (data not shown). A significant positive correlation between *TERT*-p mutation and DFS was confirmed by the univariate Cox regression analysis (hazard ratio 0.10, 95% CI 0.01–0.82). However, in the multivariate Cox regression analysis, *TERT*-p mutation status was not an independent factor affecting DFS (Table 4).

**Table 4:** Disease-free survival univariate and multivariate Cox regression analyses in 34 patients with penile squamous cell carcinoma

Variable	Disease-free survival			
	Univariate		Multivariate	
	HR (95% CI)	*p *value	HR (95% CI)	*p *value
Histologic grade (WD vs M to PD)	3.01 (0.37–24.20)	0.301		
Stage (I, II vs III, IV)	29.37 (3.58–240.87)	0.002*	179.50 (5.37–5997.0)	0.004*
Subtype (HPV vs non-HPV-related)	0.50 (0.13–1.89)	0.311		
LV invasion	6.02 (1.24–29.25)	0.026*	0.05 (0.0–0.94)	0.046*
Perineural invasion	4.39 (1.17–16.46)	0.028*	3.65 (0.66–20.13)	0.137
Necrosis	4.67 (1.17–16.46)	0.031*	2.33 (0.46–11.77)	0.308
Mitotic figure (≤ 8 vs > 8 (/HPF))	2.80 (0.74–10.55)	0.128		
MIB-1 LI (≤ 40 vs > 40)	1.99 (0.53–7.50)	0.308		
p16^INK4a^ positivity	2.70 (0.71–10.28)	0.147		
*TERT*-p (wild vs mutant)	0.10 (0.01–0.82)	0.032*	0.37 (0.03–4.10)	0.417

*HR* hazard ratio, *CI* confidence interval, *WD* well differentiated, *M to PD* moderately to poorly differentiated, *HPF* high-power field, *LV* lymphovascular, *LI* labeling index, *HPV* human papilloma virus

*p < 0.05

## Discussion

We here provide the first report of *TERT*-p mutation and its clinicopathologic significance in penile SCC. *TERT*-p mutations were detected at a high frequency (48.6%) in penile SCC, which, to our knowledge, are the most frequent mutations in penile SCC described to date (Ferrandiz-Pulido et al. 2015). Indeed, the majority of recurrent mutations reported in penile SCC are of low incidence (less than 10%) (Ferrandiz-Pulido et al. 2015; Wang et al. 2019). Notably, out of 18 cases with *TERT*-p mutations, 14 were c.-146 C > T and 3 were c.-124 C > T. These are known as mutation hotspots that have been reported to be the most common recurrent mutations in various organs and tend to be mutually exclusive, with two exceptions in which both mutations were identified in two cases of breast cancer (Huang et al. 2013). The remaining *TERT*-p mutation detected in our study was c.-124C > A, which was found in both an excision and penectomy specimen of the same patient and was the only case of sarcomatoid carcinoma in our cohort. Histologically, the transitional area from conventional SCC to sarcomatoid carcinoma was included in this sarcomatoid carcinoma case. The c.-124C > A mutation was previously reported in one mammary phyllodes tumor, as well as in urothelial carcinoma, hepatocellular carcinoma, and glioma (Yoshida et al. 2015).

The mutation rate of *TERT*-p in cutaneous SCC, which was used as a positive control, is concordant with previous reports (Griewank et al 2013). Cheng et al. evaluated *TERT*-p mutations in SCCs from different anatomic sites, finding a mutation rate of 70% for both skin and urinary bladder SCCs, but only 20% for head and neck SCC, and no *TERT*-p mutations were identified in uterine cervix and lung cancer (Cheng et al. 2015). The authors suggested that this finding supports a hypothesis of different carcinogenesis mechanisms of SCCs from different anatomic sites. Penile SCC shows a similar frequency of *TERT*-p mutations to that reported for cutaneous SCC or urothelial cancer.

ETS transcription factors, whose binding sites are generated within the promoter region through *TERT*-p mutations, are upregulated by the mitogen-activated protein kinase (MAPK) pathway; therefore, *TERT*-p mutations may be associated with mutations in genes involved in the MAPK pathway (Horn et al. 2013). Indeed, tumors harboring *TERT*-p mutation at high frequency, such as melanoma, thyroid papillary carcinoma, and glioma, are also well known for a high frequency of *BRAF* mutation. Furthermore, significant coexistence of *TERT*-p and *BRAF* mutations, and their associations with adverse clinicopathological factors have been reported in some tumors, including papillary thyroid carcinoma and melanoma, suggesting that these coexisting mutations reflect a unique mechanism to upregulate the expression of TERT, cooperatively contributing to the aggressiveness of these tumors (Macerola et al. 2015; Xing et al. 2014). Likewise, we speculated that other mutations might coexist and act cooperatively with *TERT*-p mutations in penile SCC.

There have been only a few studies conducted on genetic alterations in penile SCC, including data obtained through whole-exome sequencing (Ferrandiz-Pulido et al. 2015; Wang et al. 2019). In general, the detection rates are low in all cases (detection rates of 3–9%), the main mutated genes identified in penile SCC include *KRAS, HRAS, NRAS*, and *PIK3CA*, which are involved in the MAPK pathway (Ferrandiz-Pulido et al. 2015; Silva Amancio et al. 2017; Spiess et al. 2016; Wang et al. 2019). However, the mutational status of *TERT*-p was not investigated in that cohort (Ferrandiz-Pulido et al. 2015). Therefore, further studies on mutations coexisting with *TERT-*p and their significance are needed to obtain a deeper understanding of the roles of *TERT*-p mutations in penile carcinogenesis.

Penile SCCs are pathologically divided into two groups, HPV-related and non-HPV-related. The key oncogenic mechanism of HPV in humans is its ability to reactivate telomerase, which largely involves the E6 protein of HR-HPV. E6 protein directly binds to hTERT and telomeric DNA or participates in the epigenetic and post-transcriptional regulation of hTERT (Katzenellenbogen 2017a, b). In contrast, E7 protein can maintain telomere length by the alternative lengthening of the telomerase pathway, irrespective of hTERT. A particularly interesting finding of this study was the much higher frequency of *TERT*-p mutation in non-HPV-related type of penile SCC, based on both histologic classification and p16^INK4a^ immunoreactivity. Although we did not investigate the presence of HPV DNA, p16^INK4a^ immunostaining has been established and widely used as a surrogate marker for transcriptionally active HR-HPV (Aumayr et al. 2013; Cubilla et al. 2011). *TERT*-p mutation was found in only 1 of 12 cases with p16^INK4a^ positivity. In contrast, 14 of the 22 (63.6%) cases showing negative immunoreactivity to p16^INK4a^ harbored a *TERT*-p mutation. Similarly, the presence of differentiated PeIN, which is known to be a non-HPV-related type of PeIN in the adjacent mucosa, also correlated with *TERT*-p mutation in this study. These results suggest two major pathogenetic pathways of penile SCC that differ not only with respect to the relation to HPV but also with respect to the underlying molecular mechanism, with different mechanisms of telomerase activation. We speculate that in HPV-related penile SCC, telomerase is activated by HPV E6 in the absence of *TERT*-p mutation, whereas mutations of *TERT*-p might play a role in the mechanism of telomerase activation in non-HPV-related penile cancer.

Based on this assumption, *TERT*-p mutation is suggested as a new potential therapeutic target in non-HPV-related cancer. Although penile cancer is typically divided into two distinct groups according to clinicopathologic features and its relation with HPV, there is no difference in the treatment of these different types, which is mainly due to the low prevalence and limited data to understand the detailed mechanisms for targeted therapy. In addition, HPV-related cancer is known to respond better to radiation or chemoradiation therapy and shows a more favorable clinical course than non-HPV-related cancer (Eich et al. 2020). Therefore, further studies are needed to validate our assumption and develop targeted therapies for penile carcinoma, especially for the non-HPV-related type.

As mentioned above, several previous studies have shown associations between *TERT*-p mutations and adverse clinicopathologic parameters or poor prognosis in various types of human cancers, including thyroid papillary carcinoma, melanoma, bladder cancer, and glioma (Chen et al. 2014a; Griewank et al. 2014; Lee et al. 2015; Nasirden et al. 2016; Simon et al. 2015; Wang et al. 2014), which was the main motivation for the present study. However, in contrast to these previous reports, we found a correlation between *TERT*-p mutation with favorable clinicopathologic parameters in penile SCC, including a low histologic grade, low mitotic count, absence of necrosis, and low MIB-1 index. These results are in line with the fact that *TERT*-p mutations were more frequently detected in non-HPV-related tumors, which are generally low-grade tumors with low mitotic activities (Steinestel et al. 2015). Similarly, the tumors in non-HPV-related penile SCC tend to be larger than those in the HPV-related type, which may explain the correlation between *TERT*-p mutant type and larger tumor size. Notably, lymph node or distant metastasis rarely occurred in patients with *TERT*-p mutant type of penile SCC; however, further studies in a larger cohort will be needed to validate this result. In addition, these patients had a significantly longer DFS than patients without *TERT*-p mutation based on Kaplan–Meier and log-rank analysis. In the univariate Cox proportional hazard model, the presence of *TERT*-p mutation was a significant factor for predicting longer DFS, but this significant effect was not maintained in the multivariate analysis. Instead, clinical stage and lymphovascular permeation were identified as independent predictors for DFS. *TERT*-p mutant type, although not significant, accounted for lower stage tumors in a large proportion of our cohort and hence could be one of the confounding factors for the determination of DFS.

Given the very low incidence of penile carcinoma, this study represents a relatively thorough analysis; nevertheless, the limitation of this study is its relatively small sample size, which can be a source of potential bias. Therefore, further investigations in larger cohorts of patients and in vitro studies are needed to validate our results. Our study shows that *TERT*-p mutations are far more frequent in non-HPV-related penile SCC based on both histological classification and p16^INK4a^ immunopositivity, and offer insight into the pathogenetic mechanisms of this rare disease.

## Supplementary Information

Below is the link to the electronic supplementary material.

Supplementary file1 (PDF 870 KB)

Supplementary file2 (PDF 607 KB)

Supplementary file3 (PDF 451 KB)

Supplementary file4 (PDF 124 KB)

## Data Availability

The datasets used and/or analyzed during the current study are available from the corresponding author on reasonable request.
