# Supplementary material for: TERT promoter mutations in penile squamous cell carcinoma: high frequency in non-HPV-related type and association with favorable clinicopathologic features
Source: J Cancer Res Clin Oncol. 2021 Feb 26;147(4):1125–35. doi: 10.1007/s00432-021-03514-9 (PMC7954710; doi:10.1007/s00432-021-03514-9)
Supplement: Supplementary file 1 — Supplementary file1 (PDF 870 KB) [file 432_2021_3514_MOESM1_ESM.pdf]

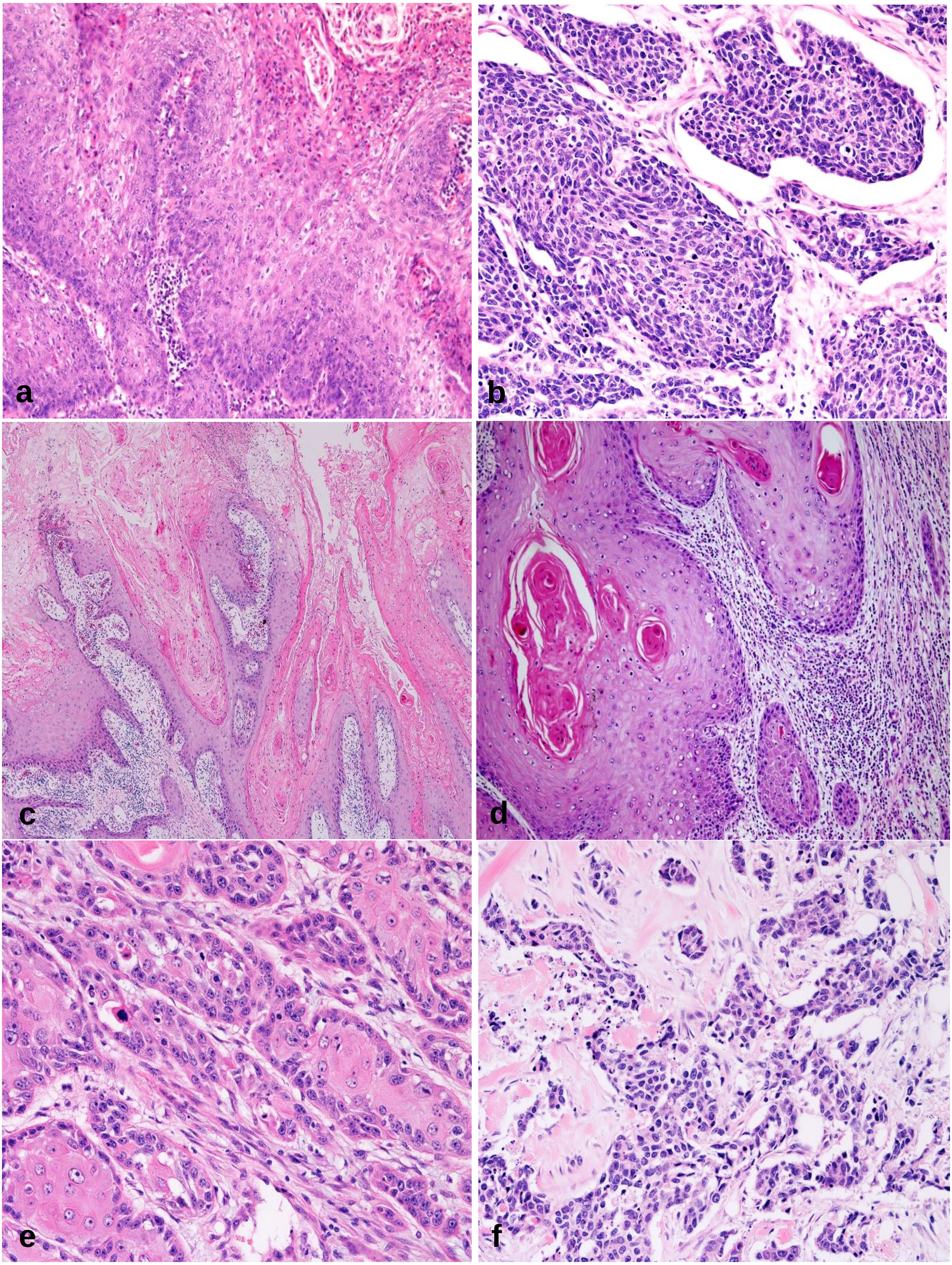

Supplementary Fig. 1 Representative images of histologic parameters. (a) Warty carcinoma (HPV-related type). (b) Basaloid carcinoma (HPV-related type). (c) Papillary carcinoma (non-HPV-related type). (d-f) Histologic grade: (d)

well-differentiated, (e) moderately differentiated, (f) poorly differentiated.

Journal of Cancer Research and Clinical Oncology

TERT promoter mutations in penile squamous cell carcinoma: high frequency in non-HPV-related type and association with favorable clinicopathologic features

Sang Kyum Kim, Jang-Hee Kim, Jae-Ho Han, Nam Hoon Cho, Se Joong Kim, Sun Il Kim, Seol Ho Choo, Ji Su Kim, Bumhee Park, Ji Eun Kwon*

***Correspondence:** Ji Eun Kwon, M.D., Ph.D.

Department of Pathology, Ajou University School of Medicine

164, Worldcup-ro, Yeongtong-gu, Suwon, 16499, Korea

E mail: kjefullup@aumc.ac.kr
