# Supplementary material for: TERT promoter mutations in penile squamous cell carcinoma: high frequency in non-HPV-related type and association with favorable clinicopathologic features
Source: J Cancer Res Clin Oncol. 2021 Feb 26;147(4):1125–35. doi: 10.1007/s00432-021-03514-9 (PMC7954710; doi:10.1007/s00432-021-03514-9)
Supplement: Supplementary file 4 — Supplementary file4 (PDF 124 KB) [file 432_2021_3514_MOESM4_ESM.pdf]

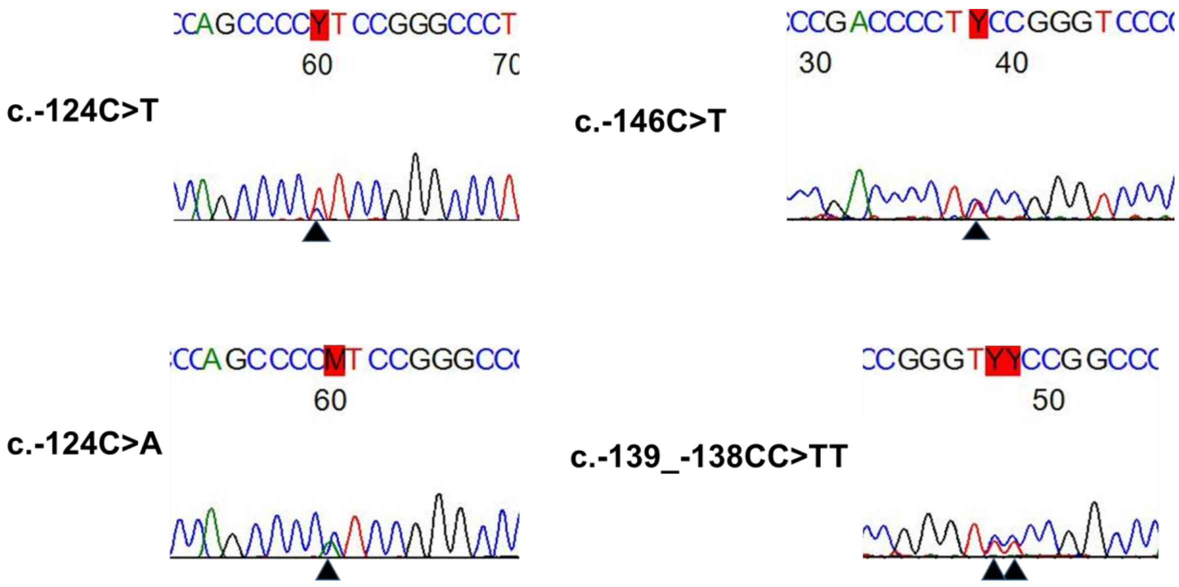

Supplementary Fig. 4 Representative images of Sanger sequencing of *TERT*-promoter mutations in penile squamous cell carcinoma and cutaneous squamous cell carcinoma.

Journal of Cancer Research and Clinical Oncology

TERT promoter mutations in penile squamous cell carcinoma: high frequency in non-HPV-related type and association with favorable clinicopathologic features

Sang Kyum Kim, Jang-Hee Kim, Jae-Ho Han, Nam Hoon Cho, Se Joong Kim, Sun Il Kim, Seol Ho Choo, Ji Su Kim, Bumhee Park, Ji Eun Kwon*

***Correspondence:** Ji Eun Kwon, M.D., Ph.D.

Department of Pathology, Ajou University School of Medicine

164, Worldcup-ro, Yeongtong-gu, Suwon, 16499, Korea

E mail: kjefullup@aumc.ac.kr
